# Membranophone percussion instruments in music therapy with adult patients in the health context: a scope review*

**DOI:** 10.1590/1980-220X-REEUSP-2022-0263en

**Published:** 2023-07-21

**Authors:** Renata Souza Souto Tamiasso, Vladimir Araujo da Silva, Ruth Natalia Teresa Turrini

**Affiliations:** 1 Universidade de São Paulo, Escola de Enfermagem, Departamento de Enfermagem Médico-Cirúrgica, São Paulo, SP, Brazil.; 2 Universidade Federal de Santa Catarina – Campus de Curitibanos, Coordenadoria Especial de Biociências e Saúde Única, Curitibanos, SC, Brazil.; 3 Escola de Enfermagem – Programa de Pós-graduação Enfermagem na Saúde do Adulto, Universidade de São Paulo, São Paulo, SP, Brazil.

**Keywords:** Complementary Therapies, Acoustic Stimulation, Music Therapy, Nursing, Review Literature as Topic, Terapias Complementarias, Estimulación Acústica, Musicoterapia, Enfermería, Literatura de Revisión como Asunto, Terapias Complementares, Estimulação Acústica, Musicoterapia, Enfermagem, Literatura de Revisão como Assunto

## Abstract

**Objective:**

To map scientific knowledge about the use of percussion instruments in music therapy in individuals over 18 years of age in the health context.

**Method:**

Scope review with search strategy implemented in September 2021, in 13 databases, using indexed descriptors and keywords. Studies on the use of membranophones for care of people over 18 years of age were included. Studies with the participation of pregnant women, psychiatric patients (schizophrenia, psychosis, addiction), or people with hearing impairment, and journal editorials were excluded. The selection process was carried out by two independent researchers.

**Results:**

Thirteen studies were included and the results showed that the membranophones have a positive impact on the physical, psychological, and social health of people in different care environments, and allow them to repeat rhythmic patterns and play music. Active music therapy was the strategy predominantly used in interventions, and the most used membranophone was the djembe.

**Conclusion:**

The results suggest that music therapy with membranophones proved to be a viable intervention with beneficial results in improving physical, psychological, and social health of people over 18 years of age.

## RESUMO

**Objetivo:**

Mapear o conhecimento científico sobre o uso de instrumentos de percussão na musicoterapia, em indivíduos maiores de 18 anos, no contexto de saúde.

**Método:**

Revisão de escopo com estratégia de busca implementada em setembro de 2021, em 13 bases de dados, utilizando-se descritores indexados e palavras-chave. Foram incluídos estudos sobre o uso de membranofones no cuidado a pessoas maiores de 18 anos. Foram excluídos estudos com participação de gestantes, pacientes psiquiátricos (esquizofrenia, psicose, adição) ou pessoas com déficit auditivo; e editoriais de revistas. O processo de seleção foi realizado por dois pesquisadores independentes.

**Resultados:**

Treze estudos foram incluídos e os resultados mostraram que os membranofones impactam de forma positiva na saúde física, psicológica e social das pessoas, em diferentes ambientes de cuidado, além de habilitá-los a repetir padrões rítmicos e musicar. A musicoterapia ativa foi a estratégia predominantemente utilizada nas intervenções, e o membranofone mais utilizado foi o *djembê*.

**Conclusão:**

Os resultados sugerem que a musicoterapia com membranofones mostrou ser uma intervenção viável com resultados benéficos na melhora da saúde física, psicológica e social em pessoas maiores de 18 anos.

## RESUMEN

**Objetivo:**

Mapear el conocimiento científico sobre el uso de instrumentos de percusión en musicoterapia en mayores de 18 años en el contexto de la salud.

**Método:**

Revisión de alcance con estrategia de búsqueda implementada en septiembre de 2021, en 13 bases de datos, utilizando descriptores indexados y palabras clave. Se incluyeron estudios sobre el uso de membranófonos en el cuidado de personas mayores de 18 años. Se excluyeron los estudios con la participación de mujeres embarazadas, pacientes psiquiátricos (esquizofrenia, psicosis, adicción) o personas con discapacidad auditiva; editoriales de revistas. El proceso de selección fue realizado por dos investigadores independientes.

**Resultados:**

Se incluyeron trece estudios y los resultados mostraron que los membranófonos tienen un impacto positivo en la salud física, psicológica y social de las personas en diferentes entornos de atención, además de permitirles repetir patrones rítmicos y reproducir música. La musicoterapia activa fue la estrategia predominantemente utilizada en las intervenciones, y el membranófono más utilizado fue el djembe.

**Conclusión:**

Los resultados sugieren que la musicoterapia con membranófonos demostró ser una intervención viable con resultados beneficiosos en la mejora de la salud física, psicológica y social de las personas mayores de 18 años.

## INTRODUCTION

In Brazil, Health Complementary Integrative Practices (*PICS*) are accessible to the population through the National Policy on Integrative and Complementary Practices (*PNPIC*), in the Brazilian Public Health System (*SUS*), legitimized by Ordinance No. 971, of May 3, 2006^(1)^, following the guidelines of the World Health Organization (WHO). Currently, the *PNPIC* has 29 health *PICS*, including music therapy^(2)^.

Music therapy produces several effects in patients, such as relaxation, distraction, stress relief, decrease in anxiety, pain, fatigue, and improvement of depression symptoms. It is also related to patient satisfaction and contributes to heart rate, respiratory rate, and blood pressure regulation^(3)^.

Besides the diversity of situations in which music therapy can be applied with beneficial effects for the individual, it can be used in the treatment of patients of all ages: newborns, children, adolescents, adults, and the elderly^(4,5,6,7)^.

Music therapeutic effect has been explained based on three aspects: cognition, emotion, and neurobiology. Musical stimuli have a biological effect on behavior by participating in specific brain functions involved in memory, learning, and multiple motivational and emotional states. Auditory perception of music occurs in the auditory center of the brain’s temporal lobe, which sends signals to the thalamus, midbrain, pons, amygdala, medulla, and hypothalamus^(8)^.

Music therapy can be performed with patients basically in two ways: actively or passively (receptive). Engagement with music is passive when the intervention only includes listening by the patient, either through headphones or ambient music, performed live by a professional or brought by him/her on CD, radio, iPod, or computer. The production of music by the patient (singing, playing an instrument) can be called active music therapy^(9)^.

Some musical instruments, such as percussion instruments, have the main function of highlighting the music rhythm. Some of them, according to the Hombostel-Sachs classification, are called membranophones^(10)^. In membranophones, sounds are produced primarily by the vibration of an extended and tensioned membrane on a given support, which can be made of skin, fabric, or synthetic material^(11)^, such as drums, *tamborim*, *pandeiro*, *surdo*, djembe, and bass drum.

The use of the membranophone in clinical practice involves music and movement according to the rhythm. The effect of the interventions is linked to the objectives and outcomes determined by the researcher. A controlled clinical trial with an oncology nurse observed, after a month of weekly music sessions with exercises and music, a reduction in depression, anxiety, and psychosomatic symptoms compared to the control group^(12)^. A study on the effect of music interventions with drums on the well-being of users and professionals of mental health services identified, through interpretive phenomenological analysis, hedonia, proactivity, and greater ability to act according to one’s own will and freedom to make choices, sense of accomplishment by being able to participate in activities and group identity, better focus and concentration, greater self-perception and self- awareness, social well-being, and belonging^(13)^.

Scope review on the use of music therapy to promote health, improve quality of life and functionality in military personnel noted that most studies with clinical purposes used active music and almost all of these with membranophones. Studies with drums identified reduced loneliness, access to memories, greater group cohesion, greater self-control and mood control, better communication and expression of emotions^(14)^.

Systematic reviews of studies with music therapy in care practice are frequent, but few with active music and the use of membranophones, which justifies the relevance of this study, whose objective was to map scientific knowledge about the use of percussion instruments in music therapy in individuals over 18 years of age in the health context.

## METHOD

The scope review model proposed by the JBI model^(15)^ was used and the recommendations of the PRISMA ScR declaration^(16)^ with the following steps: definition and alignment of objectives to the research question, development of the inclusion criteria consistent with the objectives and the research question, description of the plan to search for evidence, selection, data extraction, search and selection of evidence, extraction and analysis of evidence, results, summary of evidence focused on the purpose of the review, conclusions, and implications of findings^(15)^.

This review was guided by the following question: “Which are the investigations available in the literature on the use of percussion instruments such as membranophones in the context of health in people aged over 18?”

### Data Sources and Research Strategy

**Chart 1:**
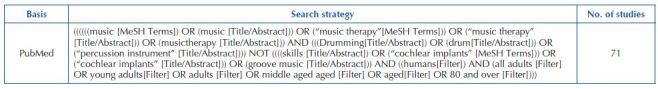
Search strategy in English used in one of the databases. São Paulo, Brazil, 2021.

The search structure considered as population (P), people ≥18 years old; the concept (C), music therapy with membranophone-type percussion instruments; and the context (C), health context (hospital, outpatient’s department, community, home, long-stay institutions, primary health units). The search was carried out with the help of a librarian in September 2021, using indexed descriptors and keywords (Supplementary Material Table 1), in the databases: Virtual Health Library (VHL), *The Cumulative Index to Nursing and Allied Health Literature* (CINHAL), Cochrane Library, *Excerpta Medical Database* (EMBASE), SCOPUS, Epistemonikos, *JBI Library*, Prospero, PsychINFO, PUBMED (Chart 1), SciELO, *Science Direct, Scopus* and *Web of science*. Filters were used (humans, adults, and age groups of interest) when the portal had this resource and option for words in the title or abstract. The preliminary search on the subject allowed including the Boolean term NOT to reduce the capture of studies on cochlear implants, *groove music* (which awakens good feelings and desire to dance), and musical skills. In the selection process, gray literature material was accessed in the ETHOS and CAPES Theses and Dissertations databases.

### Inclusion and Exclusion Criteria

Studies without time frame in Spanish, French, English, Italian, and Portuguese, which made use of music with membranophone in people over 18 years of age in the context of health were inclusion criteria. Studies with the participation of pregnant women, psychiatric patients (schizophrenia, psychosis, addiction), or people with hearing impairment, and journal editorials were excluded.

### Selection of Studies

The selection of studies was made manually in the software Excel and by two independent reviewers who assessed the title and abstracts of potentially relevant studies using the selection criteria. A third reviewer was consulted in case of disagreement about the eligibility of the document. Eligible articles were analyzed in full.

### Data Extraction

Data were extracted using the *JBI template source of evidence details, characteristics and results extraction instrument* combined with a complementary instrument specific to the purpose of the investigation based on the reporting guidelines for music interventions (Supplementary Material Table 1). The extracted data included: author, year of publication, country of origin, journal, description of populations (sex, age), number of participants, type of groups (intervention, control and placebo), objective, methodology of study, ethical approval, musical intervention time, type of delivery (recorded *versus* live music, description of the intervention, comparator and details thereof, assessment instruments, significant results for the purpose of the review and main conclusions that relate to the review question.

### Data Synthesis

The synthesis of the main aspects related to the intervention and its results were presented in summary charts. When relevant, descriptive statistical measures were used to group the information.

## RESULTS

**Figure 1:**
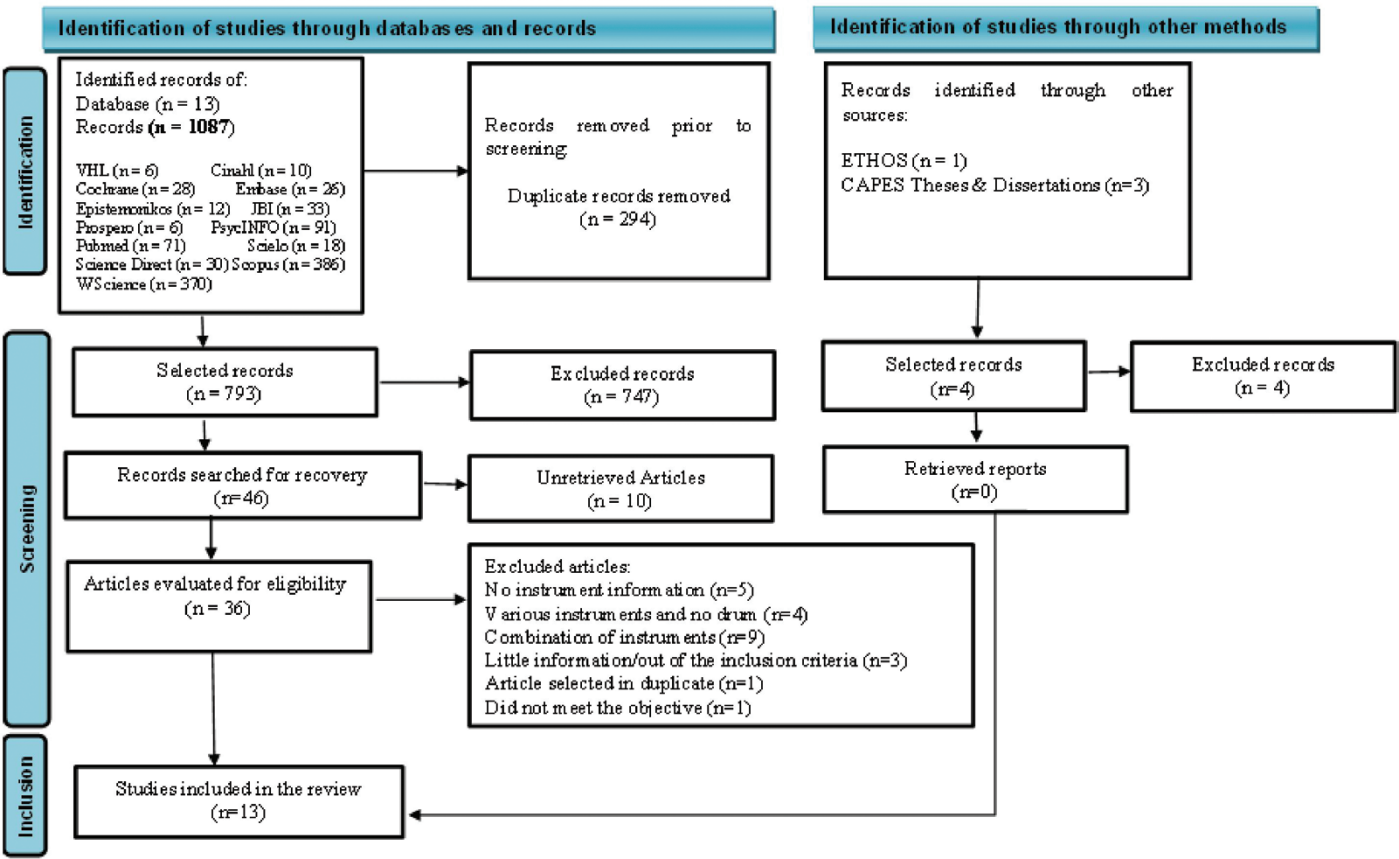
PRISMA-ScR flow diagram of the process of searching and selecting the studies for the review.

A total of 1,087 studies were retrieved and after eliminating duplicates using Excel spreadsheets, 793 remained. After reading the title and abstracts, 747 were eliminated for addressing child populations, musical skills, music theory, clinical complications arising from the use of certain membranophones, student activities, musical styles, and use of a variety of instruments. Of the remaining 46, some were excluded because they were unable to access the full text (n = 3) or because they were clinical trial protocols (n = 4) and conference abstracts (n = 3). Therefore, the full texts of 36 studies were read and 23 were excluded due to lack of information on the musical instrument used (n = 5), use of other musical instruments (n = 4), use of membranophones with other instruments (n = 9), review studies with limited information about the intervention or within the exclusion criteria (n = 3), article selected in duplicate (n = 1) or that did not meet the objective of the review (n = 1). The final sample consisted of 13 articles (Figure 1).

**Chart 2:**
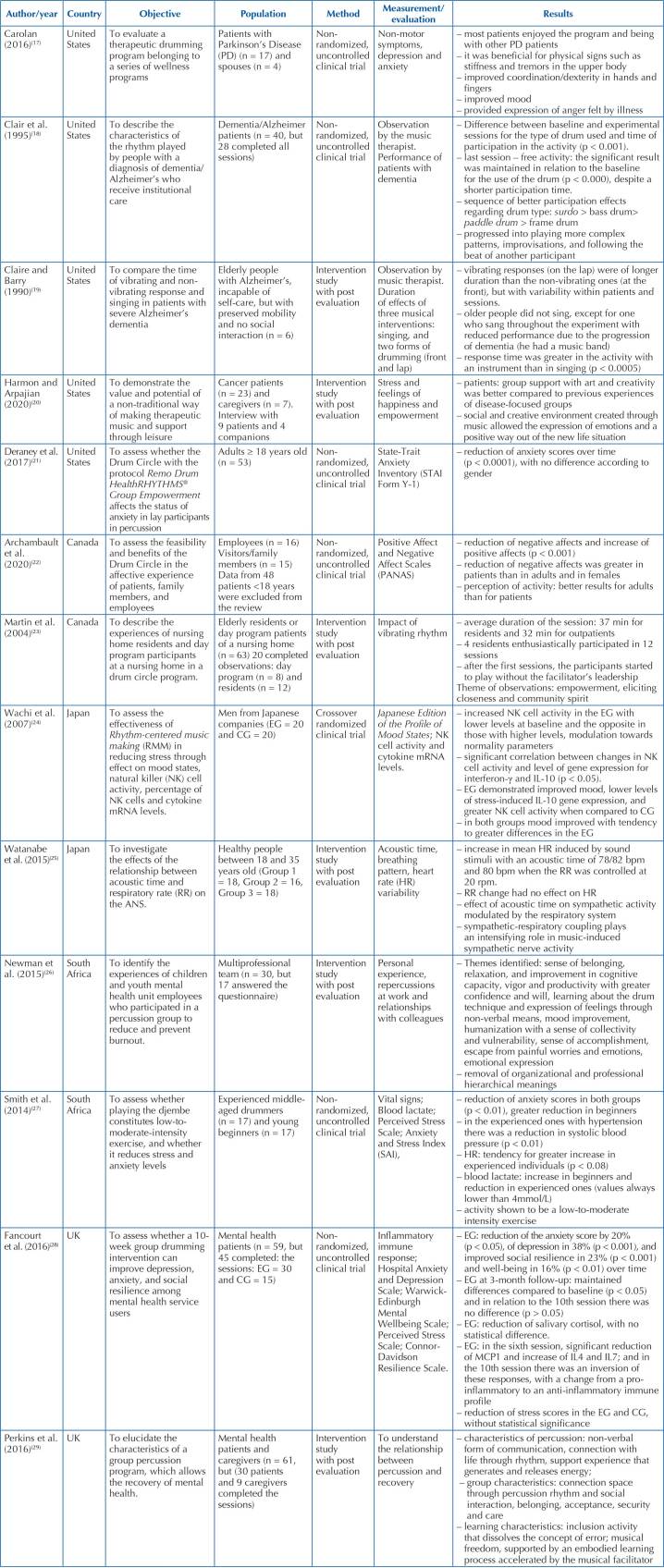
Publications included in the scoping review by author/year, country, objective, population, method, measurement/evaluation and results. São Paulo, Brazil, 2022.

Chart 2 presents the synthesis of the studies. The studies were published between the 1990s and 2020^(17–29)^, most of them (61.5%) between 2015 and 2020^(17,20–22,25,26,28,29)^. Of the 13 studies included, five (38.4%) were published in the United States^(17–21)^, two (15.4%) in Canada^(22,23)^, two (15.4%) in Japan^(24,25)^, two (15.4%) in South Africa^(26,27)^, and two (15.4%) in the United Kingdom^(28,29)^.

EG = experimental/intervention group; CG = control group; MCP1 = monocyte chemotactic protein; IL = interleukin; bpm = beats per minute; rpm = respirations per minute.

As for the research designs, the following were included: four (30.8%) non-randomized and non-controlled clinical trials^(17,18,21,22)^, two (15.4%) non-randomized controlled clinical trials^(27,28)^, one (7.7%) crossover randomized clinical trial^(24)^, and six (46.1%) intervention studies with post evaluation^(19,20,23,25,26,29)^. Regarding the analysis method, two (15.4%) were mixed^(18,22)^, five (38.5%) quantitative^(21,24,25,27,28)^, and six (46.1%) qualitative^(17,19,20,23,26,29)^. Most of the studies carrying out qualitative analyses^(17,19,20,23,26,29)^ used interviews in their data collection, semistructured interview^(20)^, field notes, audiotaped interviews^(23)^, semi-structured questionnaire with open questions^(26)^, semi- structured individual interviews and focus group interviews^(29)^ and notes from observers^(19)^.

Interventions with membranophones showed good effects for the expression of emotions in all studies, the reduction of negative affects and the increase of positive ones^(22)^, mood improvement^(17,24,26)^, improvement of anxiety^(21,27,28)^, improvement of depression, social resilience and well-being^(28)^, improvement in upper body stiffness and tremors in patients with Parkinson’s disease^(17)^, improvement in social interaction/communication in patients with dementia^(18,19)^, empowerment in the elderly^(23)^, relaxation and improved productivity in health professionals^(26)^, reduction of systolic blood pressure in hypertensive people^(27)^. It was also found that percussion is a low to moderate intensity exercise^(27)^ and it was concluded that the effect of acoustic time on sympathetic tone is modulated by the respiratory system^(25)^.

While one study did not specify the location of the intervention^(29)^, for the others, the interventions were implemented in a pediatric hospital^(22)^, National Parkinson Foundation Center of Excellence at a teaching hospital^(17)^, long-stay institution^(18,19,23)^, hospital^(21)^, clinic^(20)^, psychiatric hospital^(26)^, rented space near the residence of the experimental group participants^(28)^, space for corporate events^(27)^, *Yamaha Health Management Center* ^(24)^, and soundproofed listening room^(25)^.

The studies used structured protocols for intervention with membranophones, such as the “F*ind your beat”* of *Health Rhythms* ^*TM*^ *Group Empowerment Drumming* ^(17)^. Two studies also followed the training of the *Health Rhythms* ^*TM*^ *Group Empowerment Drumming*, but they did not name the protocol^(21,24)^. Ten studies presented the description of the steps of the intervention protocol^(19–21,23–29)^. One of the articles did not present the described protocol, but mentioned that it was the reproducibility of a protocol already used^(18)^. Another study applied the protocol *Health Rhythms* ^*TM*^ as a basis, adapting it according to the participants’ responses over the course of the intervention weeks^(17)^.

As for the music therapy approach, 12 studies (92.3%) mentioned that the patients received active music therapy^(17–24,26–29)^ and only one (7.1%) used passive music therapy^(25)^, but it was a study to assess how the effect of rhythm on the autonomic nervous system occurs.

**Chart 3:**
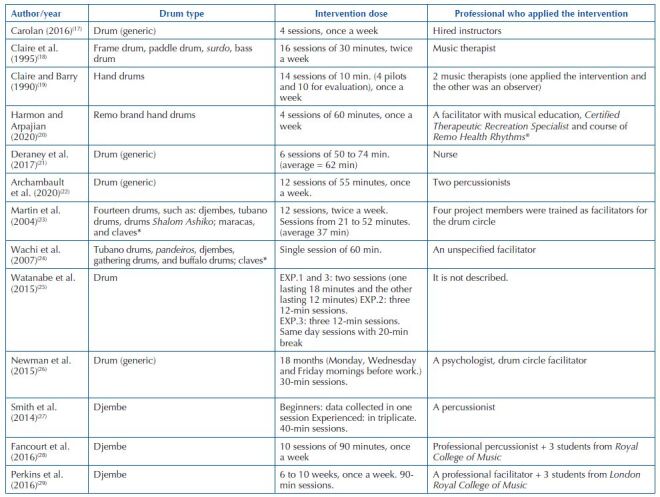
Intervention characteristics according to type of membranophone, intervention dose and professional who applied the intervention. São Paulo, Brazil, 2022.

Regarding the intervention characteristics (Chart 3), it was possible to observe that of the 13 studies, only one (7.7%) did not report the time of exposure to the intervention^(17)^; the others presented information that was synthesized by measures of central tendency and variability, with a mean time of 48.1 (±24.9) minutes, a median of 47.5 minutes and modes of 30, 60 and 90 minutes being obtained. The variability between the number of sessions was wide, from one to 14 sessions. The study mentioning 18 months was excluded from this group because the program was continuous and with free participation^(26)^, as well as the analytical study carried out in the hearing laboratory in a single day^(25)^.

*Whether any participant chose the idiophone was not mentioned (apparently not).

The most used membranophone in the studies was the djembe, and in three studies (23.1%) it was exclusively used^(27–29)^ and in two (15.4%) studies it was used with other membranophones^(23,24)^. Some studies referred to drums by Remo, which is a drum skin company.

Although two studies mentioned providing the group with one^(24)^ or two^(23)^ idiophones, they were kept in the analysis because the participants were free to choose the instrument in the sessions and the selection of this type of instrument was not made explicit. The protocols used only mentioned membranophones. 

Data were extracted following the recommendations of the reporting guidelines for music-based interventions^(30)^. Among the seven items that compose them, only two (intervention theory and music) were not satisfactorily reported by the studies (Supplementary Material Table 2).

## DISCUSSION

This scope review allowed the mapping of scientific knowledge about the therapeutic use of percussion instruments such as membranophones in people over 18 years of age in the health context. The healing and restorative power of music can be attributed to the ubiquitous social qualities in consuming and making music, which are essential for life course development, particularly for those who appreciate music^(20,31)^.

Regarding the physiological effects, it was found that music therapy with membranophone-like percussion instruments positively influences heart rate, respiratory rate, and blood pressure. Other music therapy studies corroborate these results^(32,33,34)^. As for the immune response, there was improvement, similarly to what was evidenced in other studies^(35,36)^.

Psychological effects were also observed, both in patients and in the multidisciplinary team. These findings are related to the improvement of the following aspects: positive affect, stress, anxiety, and mood. These results are also corroborated by other studies^(32,34,35,37,38)^.

The musical intervention provided a better way to face cancer treatment. This experience with art and creativity was better than previous individual or group experiences focusing on the disease. Moreover, the social and creative environment allowed, through music, the expression of emotions and a positive way out of the new life situation^(20)^. Cancer is a chronic disease that involves complex treatments and sudden changes in life. Coping with the disease and everything that involves it is a challenging process and music therapy provides support to the patient during the cancer treatment process^(39,40,41)^.

A reasonable number of studies presented results related to the elderly population, with emphasis on the use of membranophones in a long-stay institution, as they require less cognitive demand, providing an improvement in mood and in quality of life, ability to imitate rhythm patterns that gradually become more complex^(18)^. These findings are validated by other studies showing positive results in the elderly population after music therapy interventions^(38,42,43,44,45)^. Another finding, inherent to the elderly population is related to the vibrating responses (when the drum was placed on the participant’s lap and the participant felt the vibration), which lasted longer than the non-vibrating responses (when the drum was held by a music therapist, in front of the participant, off the patient’s lap)^(19)^.

Playing the drum goes beyond hearing the sound, as it is possible to feel the sound through vibration. This refers to the first sounds heard and felt by the baby in the mother’s womb, the heartbeat^(46)^, a Universal ISO such as the sounds of inspiration and expiration, the whisper of a mother’s voice, blood flow and many others that arise from nature and human beings. Universal ISO is a dynamic sound structure, which characterizes human beings, regardless of their social, cultural, historical, and psychophysiological contexts^(47)^. The rhythm is an innate and natural part of individuals; the execution of the vibrating instrumental rhythm is a positive experience, especially when performed in a group, improving communication, promoting community musical making^(23,48)^.

When considering the music therapy delivery method, most participants received active music therapy. The practice of percussing a membranophone dispenses knowledge of musical notation or writing, making its use suitable for group practice, even with great heterogeneity among its participants in relation to the level of knowledge or prior musical involvement^(29)^. The session conducted by a qualified facilitator allows the assimilation of knowledge required to play the instrument pleasantly and productively.

With diversity in weight, shape, size, cost, and raw material, membranophones are practical for transportation, execution, and acquisition. As for the type of musical instrument, the predominant membranophone in the studies was the djembe, a percussion instrument that does not have large dimensions, being found in several sizes, easy to transport and acquire considering the variability of price and access to it. Unlike other drums common in a given locality or region, the djembe is present in several countries^(32,49,50,51)^.

Musical interventions with membranophones can be performed individually or in groups. In a group, the intervention is known as “Drum Circle”. The drum circle was studied in five articles^(17,20,23,26,29)^ cited in this review. Drum circles are an ancient practice that has been part of the healing rituals of many cultures around the world since antiquity and, nowadays, has been structured as an intervention used in the health area. Some protocols were structured for application in clinical practice^(52)^. The *Health Rhythms* is a protocol used in the reviewed studies that was developed to be applied in group interventions with percussion instruments^(53)^.

### Study Limitations

Some limitations of the sample should be considered. Most of the studies included are quasi-experimental, that is, there was no randomization, and many did not include a control group, which weakens their conclusion. The limited description of interventions in some studies, the sample sizes, and methodological limitations of most of the included studies provide weak evidence on the implications of using membranophones in adult health. Only one study^(28)^ performed follow-up, where the results of the intervention persisted after three months, which highlights the need for more longitudinal and experimental randomized and controlled studies to produce better levels of evidence. Regarding this study, gray literature sources were little explored in the search (totaling two sources), where no studies were identified that represented the targeted mapping. Another limitation is the failure to search for articles of interest in the references of articles selected for this review. 

## CONCLUSION

Music therapy with membranophones showed beneficial results in improving levels of stress, anxiety, depression, mental well-being, change from pro-inflammatory to anti-inflammatory profile, and greater well-being for cancer patients. Its use was identified in various environments, such as hospitals, outpatient clinics, or long-stay institutions, in the most diverse health contexts. Furthermore, it was possible to map the diversity of drums used in the sessions: djembe, *pandeiro*, bass drum, tubano drum, *surdo*, frame drum, paddle drum, buffalo drum, hand drum, and gathering drum.

Unlike other musical instruments, most membranophones do not require practice or prior knowledge from the performer, and have proven to be a suitable instrument for group music therapy activities and socialization. This accessibility is closely related to the presence of the facilitator, who applies the protocol and, when necessary, modulates and adjusts the intervention, introducing gradual variations or changes in response to what was expressed by the subject. The role of facilitator can be performed by several duly qualified professionals, such as nurses, active care agents, who are present in different health contexts.

Given the benefits and feasibility of active music therapy with membranophones in care settings, with adults, people with cancer, healthy elderly people or those with dementia and/or Parkinson’s disease, the multidisciplinary team, visitors, and patients’ families, this review supports future studies with robust methodologies for the search for scientific evidence on the use of membranophones in therapeutic practice and nursing care.

## Supplementary Material

The following online material is available for this article:

Table 1 – Database name, search strategy, and number of retrieved articles. São Paulo City, Brazil, 2022.

Table 2 – Quality of the intervention report based on the Checklist for Reporting Music-Based Interventions. São Paulo City, Brazil, 2023.
