# Supplementary material for: Membranophone percussion instruments in music therapy with adult patients in the health context: a scope review
Source: Rev Esc Enferm USP. 2023 Jul 21;57:e20220263. doi: 10.1590/1980-220X-REEUSP-2022-0263en (PMC10364967; doi:10.1590/1980-220X-REEUSP-2022-0263en)
Supplement: Supplementary file 1 [file 1980-220X-reeusp-57-e20220263-s1.pdf]

Rev Esc Enferm USP
https://doi.org/10.1590/1980-220X-REEUSP-2022-0263en

Supplementary Material for "Membranophone percussion instruments in music therapy with adult patients in the health context: a scope review"

Table 1 - Database name, search strategy, and number of retrieved articles. São Paulo City, Brazil, 2022.

Name	Search strategy	Quantity and studies
Virtual Health Library: VHL (BIREME)	((((mh:(Music)) OR (Music) OR (mh:(Music therapy)) OR ("Music therapy ") OR (Musictherapy) OR (Therapy, music)) AND ((drumming) OR (drums) OR (percussion instrument)) AND NOT ((Skills) OR (mh:(cochlear implants)) OR (cochlear implants) OR (groove music))) AND NOT (animal)) AND NOT (child OR adolescent)	6
CINAHL	((TI music OR AB music OR TI (music therapy or music intervention or musical therapy or music-based intervention or therapeutic music) OR AB (music therapy or music intervention or musical therapy or music-based intervention or therapeutic music)) AND (TI drumming OR AB drumming OR TI drumming through music OR AB drumming through music OR TI drums OR AB drums OR TI percussion instrument OR AB percussion instrument)) NOT (TI skills OR AB skills OR TI (cochlear implant or cochlear implants or cochlear implantation) OR AB (cochlear implant or cochlear implants or cochlear implantation) OR TI grove music OR AB grove music)	10
Cochrane Database of Systematic Reviews (CDSR)	(("music" OR "music therapies" OR "music therapy" OR Musictherapy) AND (Drumming OR drum OR percussion instrument)) NOT ("cochlear-implant" OR "cochlear-implants" OR Skills OR groove music)	28
EMBASE	(((music:ab,ti OR 'music therapy':ab,ti OR musictherapy:ab,ti) NOT skills:ab,ti OR 'cochlea implants':ab,ti OR 'cochlea prosthesis':ab,ti OR 'groove music':ab,ti) AND ((drumming:ab,ti OR drums:ab,ti OR 'percussion instrument':ab,ti) NOT skills:ab,ti OR 'cochlea implants':ab,ti OR 'cochlea prosthesis':ab,ti OR 'groove music':ab,ti)) AND (([adult]/lim OR [young adult]/lim OR [middle aged]/lim OR [very elderly]/lim) AND [humans]/lim AND [embase]/lim)	26
Epistemonikos	((Musictherapy OR Music OR "Music therapy") AND (Drumming OR Drum OR "Percussion instrument")) NOT	12

	(Skills OR "cochlear implant" OR "groove music")	
JBI Evidence Synthesis	*((Music OR "Music therapy" OR "music therapies" OR Musictherapy) AND (Drumming OR drum OR "percussion instrument")) NOT (Skills OR "cochlear implant" OR "cochlear implants" OR "groove music"); ((Music OR "Music therapy" OR "music therapies" OR Musictherapy) AND (Drumming OR drum OR "percussion instrument")) NOT (Skills OR "cochlear implant" OR "cochlear implants" OR "groove music")*	33
Prospero	((Music OR Musictherapy OR "Music therapy") AND (Drumming OR drum OR "percussion instrument")) NOT (Skills OR "cochlear implant" OR "groove music")	6
PsycINFO	(((**MeSH**: (music)) *OR* (**title**: (music)) *OR* (**abstract**: (music)) *OR* (**MeSH**: ("Music Therapy")) *OR* (**title**: ("Music Therapy")) *OR* (**abstract**: ("Music Therapy")) *OR* (**title**: (Musictherapy)) *OR* (**abstract**: (Musictherapy))) *AND* ((**title**: (drumming)) *OR* (**abstract**: (drumming)) *OR* (**title**: (drum)) *OR* (**abstract**: (drum)) *OR* (**title**: ("percussion instrument")) *OR* (**abstract**: ("percussion instrument")))) *NOT* ((**title**: (Skills)) *OR* (**abstract**: (Skills)) *OR* (**MeSH**: ("cochlear implant")) *OR* (**title**: ("cochlear implant")) *OR* (**abstract**: ("cochlear implant")) *OR* (**title**: ("groove music")) *OR* (**abstract**: ("groove music"))) *AND* **Age Group**: Adulthood (18 yrs & older) *AND* **Population Group**: Human	91
PubMed (Medline)	((((((music[MeSH Terms]) OR (music[Title/Abstract])) OR ("music therapy"[MeSH Terms])) OR ("music therapy"[Title/Abstract])) OR (musictherapy[Title/Abstract])) AND (((Drumming[Title/Abstract]) OR (drum[Title/Abstract])) OR ("percussion instrument"[Title/Abstract]))) NOT ((((skills[Title/Abstract]) OR ("cochlear implants"[MeSH Terms])) OR ("cochlear implants"[Title/Abstract])) OR (groove music[Title/Abstract])) AND ((humans[Filter]) AND (alladults[Filter] OR youngadults[Filter] OR adults[Filter] OR middleagedaged[Filter] OR aged[Filter] OR 80andover[Filter]))) Filters: Humans, Adul: 19+ years, Young Adult: 19-24 years, Adult: 19-44 years, Middle Aged + Aged: 45+years, Middle Aged: 45-64 years, Aged: 65 + years, 80 and over: 80+years	71
Scielo	((((Music **OR** Music therapy **OR** Musictherapy) **AND** **(**Drumming **OR** drum **OR** percussion instrument)) **NOT (**Skills **OR** cochlear implant **OR** groove music)) NOT (animal)) NOT (child OR children PR newborn OR infant OR “preschool child” OR adolescent)	18
ScienceDirect	Title, abstract, keywords: ((Music OR "Music therapy" OR Musictherapy) AND (Drumming OR Drum OR "Percussion instrument")) NOT (Skills OR “cochlear implants” OR “groove music”) Filter: Social Sciences, Medicine and Dentistry, Psychology, Neuroscience.	30
Scopus	(((((TITLE-ABS-KEY (music) OR TITLE-ABS-KEY ("music therapy") OR TITLE-ABS-KEY ("music intervention") OR TITLE-ABS-KEY ("music based intervention") OR TITLE-ABS-KEY ("therapeutic	386

	music"))) AND ((TITLE-ABS-KEY (drumming) OR TITLE-ABS-KEY ("drumming through music") OR TITLE-ABS-KEY (drums) OR TITLE-ABS-KEY ("percussion instrument")))) AND NOT ((TITLE-ABS-KEY (skills) OR TITLE-ABS-KEY ("cochlear implant") OR TITLE-ABS-KEY ("groove music")))) AND NOT (TITLE-ABS-KEY (animals))) AND NOT (TITLE-ABS-KEY (child OR children OR newborn OR infant OR "preschool child" OR adolescent)) AND (LIMIT-TO (SUBJAREA , "soci") OR LIMIT-TO (SUBJAREA , "medi") OR LIMIT-TO (SUBJAREA , "psyc") OR LIMIT-TO (SUBJAREA , "neur") OR LIMIT-TO (SUBJAREA , "heal") OR LIMIT-TO (SUBJAREA , "mult") OR LIMIT-TO (SUBJAREA , "nurs"))	
Web of Science	**(((((music** (Tópico) or **"Music therapy "** (Tópico) or **Musictherapy** (Tópico)) AND (**drumming** (Tópico) or **drums** (Tópico) or **"percussion instrument"** (Tópico))) NOT (**skills** (Tópico) or **"cochlear implants"** (Tópico) or **"groove music"** (Tópico))) AND Articles or Conference articles or Review articles (Document types) and Music or Acoustics or Computer Science Interdisciplinary Applications or Neurosciences or Clinical Psychology or Behavioral Sciences or Geriatrics Gerontology or Nursing or Audiology Speech Language Pathology or Social Sciences Biomedical or Architecture or Area Studies or Asian Studies or Biology or Communication or Computer Science Artificial Intelligence or Education Educational Research or Ergonomics or Family Studies or Folklore or Geography or Linguistics or Medieval Renaissance Studies or Pharmacology Pharmacy or Philosophy or Sport Sciences or Medicine General Internal (Web of Science Categories) and Music or Acoustics or Psychology (Areas of Research)) **NOT ALL=(animal)) NOT TS=(child OR children OR newborn OR infant OR "preschool child" OR adolescent)**	370

Source: Prepared by the authors, 2022.

DATA COLLECTION INSTRUMENT

**A – IDENTIFICATION**	
Article title:	
Journal Title:	
Journal Classification:	
Country:	Language:
Year:	
**B- INSTITUTION OF THE STUDY**	
( ) Hospital ( ) University ( ) Research center	( ) Single institution<br>( ) Multicentric Research
( ) Other Institutions. Which one?	
**C- AUTHORS' KNOWLEDGE AREA**	
( ) Nursing ( ) Medical ( ) Psychology ( ) Mixed authorship<br>( ) Other health area. Which one?	
**D- METHODOLOGICAL CHARACTERISTICS OF THE STUDY**	
**1. Design of study**	
**If clinical trial answer**	
1.1 Controlled	( ) Yes<br>( ) No
1.2 Randomized	( ) Yes<br>( ) No
1.3 Blinding	( ) Single blind<br>( ) Double blind<br>( ) Triple blind<br>( ) Quadruple blind
**2. Objective or study question**:	
**3. Sample**	
3.1 Selection	( ) Random<br>( ) Convenience<br>( ) Other
3.2 Size (n)	( ) Initial<br>( ) Final
3.3 Inclusion/exclusion criteria for subjects:	
Inclusion	Exclusion
**4. Data processing**	
**5. Musical interventions performed**	
5.1 Control group:	( ) Yes<br>( ) No
5.2 Placebo group:	( ) Yes<br>( ) No
5.3 Duration of the study	

5.4 Music selection	( ) Selection by the author of the study<br>( ) Self-selection<br>( ) Other
5.5 Type of Music	( ) Classic<br>( ) Popular instrumental<br>( ) Religious<br>( ) Country<br>( ) Brazilian Popular Music<br>( ) *Axé*<br>( ) Samba<br>( ) Rock<br>( )Others. Which one?
5.6 Features of the song	
5.7 Application of the intervention	( ) Headset<br>( ) Background music<br>( ) Live music<br>( ) Other
5.8 Professional who applied the intervention	( ) Nurse<br>( ) Physician<br>( ) Music therapist<br>( ) Other
5.9 Time of exposure to the intervention	( ) 10 to 30 min<br>( ) 31 to 60 min<br>( ) 61 to 120 min<br>( ) Over 120 min
5.10 Type of percussion instrument	( ) undefined pitch Which one? _________________<br>( ) defined pitch. Which one?<br>( ) Other
2.6 Measurement instrument	( ) Yes<br>( ) No
5.12 Methods used to measure the intervention	( ) Scales Which one ________________________<br>( ) Vital signs Which one ______________________<br>( ) Laboratory Tests Which ones _______________<br>( ) Patient report<br>( ) Others
**6. Results**	
**7. Analysis**	
7.1 Statistical treatment	
7.2 Significance level	
**8. Implications**	
8.1 Conclusions are justified based on the results	
8.2 What are the authors' recommendations	
**9. Level of Evidence**	
**E- ASSESSMENT OF METHODOLOGICAL RIGOR**	
1. Clarity in identifying the methodological path in the text (method used, participating subjects, inclusion/exclusion criteria, intervention, results)	( ) Yes<br>( ) No
2. Identification of limitations or biases	( ) Yes<br>( ) No
3. Identification of conflict of interest.	( ) Yes<br>( ) No
