# Supplementary material for: Membranophone percussion instruments in music therapy with adult patients in the health context: a scope review
Source: Rev Esc Enferm USP. 2023 Jul 21;57:e20220263. doi: 10.1590/1980-220X-REEUSP-2022-0263en (PMC10364967; doi:10.1590/1980-220X-REEUSP-2022-0263en)
Supplement: Supplementary file 2 [file 1980-220X-reeusp-57-e20220263-s2.pdf]

Rev Esc Enferm USP
https://doi.org/10.1590/1980-220X-REEUSP-2022-0263en

Supplementary Material for "Membranophone percussion instruments in music therapy with adult patients in the health context: a scope review"

Table 2 - Quality of the intervention report based on the Checklist for Reporting Music-Based Interventions. São Paulo City, Brazil, 2023.

Criteria for the Music-Based Intervention Report	Yes		NO		Study
	Quantity	%	Quantity	%	
**1 Intervention theory/ Justification** (n=13)<br>Provide a rationale for the song selected; specify how the qualities and delivery of the music are expected to affect the desired results.	6	46%	7	53%	(20, 22, 24-25, 27-28)
**2 Intervention Content** (n=13)<br>Provide precise details of the music intervention and, where applicable, descriptions of procedures for tailoring interventions to individual participants.	13	100%	0	0	(17-29)
**2.1 Person who selected the music** (n=13)<br>Specify who selected the music: pre-selected by investigator, participant from a limited set, participant selected from his/her own collection, customized based on patient assessment.	13	100%	0	0	(17-29)
**2.2 Music**<br>Specify the specific source of the published/recorded music or describe the qualities of the original/improvised music.					
When using published music, please provide reference to music score or sound recording. (n=0)	-	-	-	-	-
When using improvised or original music, describe the general structure of the music (eg, form, elements, instruments). (n=13)	12*	92%	1** no	8%	(17-20, 22-29)
**2.3 Music Delivery Method (Live or Recorded).**					
When using live music, please specify. (n=12)					
Who delivered the music.	12	100%	0	0	(17-24, 26-29)
Size of acting group (eg, interventionist only, interventionist and participant).	12	100%	0	0	(17-24, (26-29)
When using recorded music, please specify. (n=1)					

Used playback equipment and/or used headphones versus speakers.	1	100%	0	0	(25)
Decibel levels of streamed music and/or use of volume controls to limit decibels.	1	100%	0	0	(25)
**2.4 Materials for the Intervention** (n=13) Specify musical and/or non-musical material.<br>Musical materials.<br>Non-musical materials.	13	100%	0	0	(17-29)
**2.5 Intervention Strategies** (n=13)<br>Describe the intervention strategies based on the music under investigation (eg listening to music, songwriting, improvisation, rhythmic auditory stimulation).	13	100%	0	0	(17-29)
**3 Intervention Delivery Schedule** (n=13)<br>Report number of sessions, session duration, and session frequency.					
Number of Sessions.	13	100%	0	0	(17-29)
Session Duration.	12	92%	1	8%	(18-29)
Session Frequency.	13	100%	0	0	(17-29)
**4 Interventionist** (n=13)					
Specify the intervenor's qualifications and/or credentials.	10	77%	3	23%	(18-23, 26-29)
Specify how many interventionists provide study conditions.	12	92%	1		(17-24, 26-29)
**5 Treatment fidelity** (n=13)<br>Describe the strategies used to ensure that treatment and/or control conditions are delivered as intended (e.g., interventional training, manual protocols, and intervention monitoring).	13	100%	0	0	(17-29)
**6 Settings** (n=13)<br>Describe where the intervention took place (ie location, level of privacy, ambient sound).					
Location	12	92%	1	8%	(17-28)
Privacy level.	0	0	13	100	
Ambient sound.	0	0	13	100	
**7 Delivery unit** (n=13)<br>Specify whether interventions were delivered to individuals or groups of individuals, including group size.	13	100%	0	0	(17-29)

Source: Robb (2018), adapted by the authors (2023). * only described the instruments ** did not describe anything, not even the instruments.
